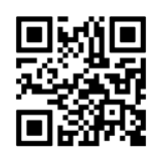# Innovative Cadaveric Technique: Utilising n-Butyl Cyanoacrylate (n-BCA) for Deep Endometriosis Excision Simulation in Minimal Invasive Surgery Training

**DOI:** 10.52054/FVVO.16.1.002

**Published:** 2024-03-28

**Authors:** M Mabrouk, S Mahgoub, A Vashisht, R Seracchioli

**Affiliations:** University College London Hospitals Foundation Trust (UCLH) & Cleveland Clinic London (CCL); Department of Gynaecology, Cambridge University Hospitals; University College London Hospitals Foundation Trust (UCLH) & Cleveland Clinic London (CCL); Gynaecology and Human Reproduction Physiopathology, Dipartimento di Scienze Mediche e Chirurgiche (DIMEC), Sant’Orsola Hospital, University of Bologna, Italy

**Keywords:** Deep infiltrative endometriosis, n-Butyl cyanoacrylate

## Abstract

**Background:**

Our study aimed to create a novel technique using n-butyl cyanoacrylate (n-BCA) for minimal access simulation training on cadavers in deep endometriosis excision.

**Objectives:**

A step-by-step video demonstration of using n-BCA in cadavers to simulate deep endometriosis. This technique is integrated into training sessions using cadavers aimed at enhancing surgical proficiency for deep endometriosis procedures.

**Material and Methods:**

Video article describing using n-BCA in cadavers as a simulation model.

**Result:**

This technique has been used in a hands-on cadaveric training course, and positive feedback supports the recommendation to incorporate this technique.

**Conclusion:**

Utilizing a human cadaver model proves beneficial for enhancing understanding of deep pelvic innervation. Implementing n-BCA in these cadaver dissections demonstrates both reproducibility and safety. This approach significantly contributes to refining surgical expertise in the excision of deep infiltrating endometriosis.

## Introduction

Learning the technique of deep endometriosis excision can be challenging due to this condition’s intricate nature and distinctive characteristics. Deep Endometriosis, also known as adenomyosis externa, typically involves lesions that penetrate more than 5 mm beneath the peritoneal surface (Gordts et al., 2017),(Wild et al., 2019). The disease can manifest in various pelvic sites, including the ovaries, pelvic peritoneum, pouch of Douglas (POD), rectum, rectosigmoid, rectovaginal septum (RVS), uterosacral ligaments (USLs), vagina, and urinary bladder. Surgical management of deep endometriosis requires advanced multidisciplinary surgical techniques, often in challenging surgical conditions, with an increased risk of complications.

Successful excision requires specialised surgical skills and a comprehensive understanding of pelvic anatomy, including precise knowledge of the location and involvement of different structures affected by endometriosis. Acquiring these skills requires a significant amount of time and extensive training.

Moreover, the diversity in disease presentation further challenges the learning process. Surgeons must adapt their approach and surgical techniques to match the specific characteristics of each patient’s disease. The ability to anticipate and recognise the extent of disease, dissect, and completely excise it while preserving pelvic nerves and organs, presents a significant challenge crucial for ensuring safe and effective surgical outcomes. Aspiring surgeons interested in mastering endometriosis excision typically undergo specialised training to address these challenges. The training may involve mentorship from experienced surgeons, participation in surgical workshops, and simulation- based learning. However, to our knowledge, there is currently no simulation module that adequately mimics the anatomical distortion often associated with deep endometriosis.

As a solution to this, we have introduced n-BCA injections during cadaver training for minimal access surgery focusing on deep endometriosis excision. This innovation allows surgeons to replicate and perform these specialised procedures in a controlled setting, enhancing their expertise and in turn, benefiting patients in clinical practice.

## Technique

The video demonstrates injecting n-BCA into the ureterovesical and rectovaginal spaces. The video emphasizes the advantages of using n-BCA, such as its ability to be injected into different retroperitoneal areas and its localised and immediate effects. Additionally, the video showcases the appearance and texture of the infiltrated area and the excision process while ensuring the preservation of surrounding structures.

In the initial part of the video, the excision and removal of the n-BCA-infiltrated areas close to the rectum are demonstrated. The subsequent part simulates a scenario where a lesion affecting the entire bladder wall is managed through a partial cystectomy.

## Discussion and Conclusion

The concept of utilising n-BCA injection draws inspiration from its application in various medical fields, such as orthopaedics for hardware fixation and plastic surgery for tasks such as blood vessel anastomoses, wound closure, skin graft application, and achieving haemostasis. Cyanoacrylate adhesives, specifically alkyl-2- cyanoacrylate monomers, are liquid and can create flexible polymers with powerful adhesive bonds to soft tissues. These adhesives, also known as cyanoacrylates, are acrylic resins that rapidly adhere and bind to surfaces in approximately one minute when exposed to water undergoing polymerisation (Ayyildiz & Ayyildiz, 2017).

After evaluating various adhesive substances, n-BCA emerged as the optimal choice due to its favourable properties. It maintains a liquid form suitable for laparoscopic injection into the retroperitoneum and quickly creates strong attachments with adjacent tissues. n-BCA remains confined to the target site, minimising the risk of dispersion to non-targeted regions, and confirming its technical superiority for such applications.

While animal models may be utilised, the cadaver model is an exemplary simulation for retroperitoneal surgery. It is particularly valuable for procedures that require careful identification and preservation of anatomical structures, such as deep with deep endometriosis. The emulation of retroperitoneal fibrosis and deep endometriosis lesions in a cadaveric setting provides a highly realistic experience for hands-on surgical training.

During the hands-on course for training in deep endometriosis excision using cadavers, we introduced the use of n-BCA infiltration in the retroperitoneal spaces. The participants provided positive feedback on their experience with excising the infiltrated n-BCA and reported it to be beneficial for their learning. This technique helped them understand retroperitoneal anatomy, identify lesions, and execute appropriate excision while preserving the surrounding structures.

Based on this positive feedback (the study is currently being published), we recommend using this technique in hands-on courses to simulate deep endometriotic lesions.

## Video scan (read QR)

https://vimeo.com/900797381/92ab9d81ba?share=copy

**Figure:**